# Manufacturing-Induced Defect Taxonomy and Visual Detection in UD Tapes with Carbon and Glass Fiber Reinforcements

**DOI:** 10.3390/polym18070807

**Published:** 2026-03-26

**Authors:** Gönenç Duran

**Affiliations:** 1 Automotive Technology Program, Department of Motor Vehicles and Transportation, Vocational School, Mudanya University, Çagrisan Campus, Bursa 16960, Türkiye; gonenc.duran@mudanya.edu.tr; 2 Mechanics and Advanced Materials Research Group Laboratory, Automotive Engineering Department, Engineering Faculty, Bursa Uludağ University, Bursa 16059, Türkiye

**Keywords:** thermoplastic PP–CF and PP–GF UD composite tapes, defect taxonomy, manufacturing-induced defects, YOLO-based visual inspection, YOLO26

## Abstract

Continuous unidirectional (UD) thermoplastic composite tapes are increasingly used in aerospace, automotive, and energy applications because of their high specific strength, low weight, recyclability, and compatibility with automated manufacturing. Since final component performance strongly depends on tape quality, reliable defect characterization and detection are essential. In this study, manufacturing-induced defects in polypropylene-based UD tapes reinforced with carbon and glass fibers were investigated using real images acquired directly from laboratory-scale production without synthetic data. Defects related to interfacial integrity, matrix distribution, fiber architecture, and surface irregularities were systematically analyzed, and a practical four-class defect taxonomy was established. To enable automated inspection under limited-data conditions, lightweight YOLOv8, YOLOv11, and the new YOLO26 models were comparatively evaluated using a UD tape-specific augmentation strategy combining physically constrained Albumentations and on-the-fly augmentation. Among the tested models, YOLO26-s achieved the best overall performance, reaching a mean mAP@0.5 of 0.87 ± 0.03, outperforming YOLOv11 (0.83) and YOLOv8 (0.78), with 0.90 precision and 0.85 recall. Interfacial (0.92 mAP) and matrix-related (0.90 mAP) defects were detected most reliably, whereas fiber-related (0.89 mAP) and surface defects (0.79 mAP) remained more challenging, particularly in glass-fiber-reinforced tapes due to transparency-masking effects. The results demonstrate the potential of compact deep learning models for computationally efficient and manufacturing-relevant in-line quality monitoring of UD tape production.

## 1. Introduction

Continuous unidirectional (UD) thermoplastic composite tapes are widely used in the aerospace, automotive, and energy sectors due to their high specific strength, impact resistance, lightweight nature, damage tolerance, recyclability, and compatibility with automated manufacturing processes. These tapes constitute the primary feedstock for advanced manufacturing technologies such as automated tape laying (ATL), automated fiber placement (AFP), and hot pressing, and the mechanical performance and structural integrity of the final components are largely governed by the quality of the tapes. Therefore, ensuring quality assurance during the production of UD thermoplastic tapes is a fundamental requirement not only for manufacturing efficiency but also for safety-critical engineering applications [1,2]

Polypropylene–carbon fiber (PP–CF) and polypropylene–glass fiber (PP–GF) based tapes offer an attractive balance between cost, processability, and performance for semi-structural and multifunctional components [3,4]. The main contribution of this study does not lie in algorithmic novelty, but rather in the systematic integration of manufacturing insight with visual inspection, providing a practical and scalable foundation for quality monitoring and process optimization in continuous thermoplastic composite tape production.

Recent studies predominantly focus on woven fabrics or fully consolidated composite components rather than continuous thermoplastic UD tapes; in other words, they mainly address the effects of defects on post-consolidation laminate structures and their inspection using image-processing-based techniques. Moreover, most vision-based approaches prioritize algorithmic performance under controlled imaging conditions and rely on large, curated datasets [1,2,3,4,5,6,7,8,9,10].

However, recent reviews indicate that production realism, limited-data scenarios, and physically interpretable defect definitions remain insufficiently addressed in many machine-learning-based inspection studies. In addition, defects associated with fiber architecture and surface-level environmental interactions pose particular challenges for vision-based inspection. Subtle fiber misalignment, dispersion, waviness, and handling-induced surface damage typically exhibit low visual contrast and gradual surface transitions, which significantly limit their detectability using conventional surface imaging techniques [5,11,12].

Research on thermoplastic automated tape laying and in situ consolidation has largely focused on the optimization of process parameters and the improvement of final laminate quality; however, a production-driven, systematic, and visually meaningful defect taxonomy for the UD tapes that constitute the input to these processes has often remained a secondary concern [13,14,15,16]. Indeed, as emphasized in the literature, the lack of standardized defect definitions and the absence of reference defect and damage datasets that realistically represent UD tapes and manufacturing environments still constitute a major barrier to the development of digital manufacturing, virtual certification, and closed-loop quality control systems for composite structures [17,18,19,20,21].

Recent studies have demonstrated that the quality of continuous unidirectional thermoplastic composite tapes is strongly governed not by the final part consolidation stage, but by production-induced defects originating during tape manufacturing. Comprehensive reviews identify common defect types such as voids, resin-rich or resin-starved regions, fiber misalignment, waviness, and interfacial debonding, and directly relate their formation to process parameters including temperature, pressure, line speed, and fiber tension throughout production [22,23]. In AFP and ATL processes where these UD tapes are employed, tape-level defects are particularly critical, as they may propagate or intensify during downstream consolidation, thereby significantly affecting the mechanical performance and structural integrity of the final components [13,14,24,25,26,27,28,29].

Focusing on such defects, Zhao et al. [21] emphasized that detecting AFP manufacturing defects through manual inspection is highly time-consuming, labor-intensive, and costly, and proposed a real-time data acquisition and online defect detection platform termed CVPS (Collection, Visualization, Post-processing System). Their system enables defect identification after each fiber layer deposition using 2D and 3D visualization, providing an intelligent alternative to conventional visual inspection practices. Similarly, Long et al. [20] investigated the necessity of real-time defect detection in an aircraft AFP production line by proposing a machine-learning-based fusion module and a single-stage defect detection approach. Their method was shown to detect and classify defects in carbon fiber-reinforced polymer components manufactured by AFP, thereby reducing reliance on manual visual inspection and improving production efficiency.

Overall, recent research indicates a growing interest in computer vision and deep learning approaches for the accurate detection of surface defects such as voids, overlaps, wrinkles, and foreign objects in AFP and ATL composite manufacturing environments [13,20,24,25]. In parallel, significant attention has been devoted to real-time industrial inspection using convolutional neural networks and to algorithmic optimizations of YOLO-based object detection architectures, aiming to enhance detection accuracy, computational efficiency, and suitability for production-scale deployment [21,29,30,31,32,33,34]. These studies highlight the need for defect classification frameworks that explicitly link manufacturing physics with visual manifestations, clarify which defect classes can be reliably detected using surface-based computer vision, and enable tape quality grading according to the intended application of the produced materials. In this context, studies that directly address how defects form during the manufacturing stage of continuous UD thermoplastic tapes, how they should be classified, and to what extent they are visually detectable remain rather limited [35,36,37,38,39,40].

In particular, research that systematically investigates the relationship between defect formation mechanisms, material-dependent optical properties, and defect visibility using image processing and deep-learning-based approaches, and that integrates such analyses into production-stage quality assessment, is still largely underdeveloped and represents a significant opportunity for further advancement [41,42,43].

This study aims to present an industrially applicable and holistic framework for the high-precision and quantitative analysis of a defect taxonomy specific to continuous PP–CF and PP–GF UD tape manufacturing, a topic that has been only sparsely addressed in the literature, by integrating You Only Look Once (YOLO)-based deep learning architectures implemented through the Ultralytics framework [44] with advanced image processing and computer vision techniques. The focus is placed on the UD tape production stage itself—that is, on the raw material prior to the AFP process—in order to investigate both defect formation mechanisms and the visual detectability of the proposed defect taxonomy.

Rather than emphasizing system-level integration, the primary objective of this work is to examine how reinforcement type and optical contrast govern defect visibility and influence the performance of YOLO-based detection. Within this framework, the study addresses two fundamental and interrelated gaps in the literature: (i) the image-based detection of a systematic defect taxonomy that is consistent with the manufacturing physics of continuous thermoplastic UD tape production, and (ii) the quantitative and experimental assessment of the visual detectability of different defect classes under realistic and limited inspection conditions.

Accordingly, continuous unidirectional PP–CF and PP–GF thermoplastic composite tapes were manufactured at laboratory scale, and the characteristic defects arising during production were systematically investigated using cost-effective image processing approaches. The resulting observations were combined with a low-computational-cost YOLO-based object detection framework, providing a physically grounded and practically feasible visual inspection strategy for early-stage quality monitoring in UD tape manufacturing.

## 2. Materials and Methods

The focus of this study is not a direct performance comparison between carbon and glass fiber-reinforced tapes, but rather the identification and visual characterization of defect classes that arise during continuous thermoplastic tape production. Furthermore, exact fiber volume fractions were not the primary control variable, as the primary focus was on visually observable production-observable defects rather than mechanical performance optimization. In this context, it was designed to represent practical and industrially suitable thermoplastic composite tape configurations.

### 2.1. Materials

The continuous unidirectional thermoplastic composite PP–CF and PP–GF tapes investigated in this study were manufactured under controlled conditions at the UMIMAG Laboratory of Bursa Uludağ University to represent thermoplastic UD tape systems commonly used in lightweight structural applications. The fiber spreading and UD tape systems developed within the scope of the TÜBİTAK-ARDEB-1005 program (Project No: 118M571) was employed.

Visual inspection during and after production revealed recurring defects related to fiber spreading and alignment, matrix impregnation quality, interface bonding, and surface-level environmental effects. Accordingly, both material systems were addressed within a unified defect taxonomy and control framework. The examined PP–CF and PP-GF UD tape production was selected to represent practical and industrially meaningful thermoplastic composite tape configurations rather than optimized material formulations.

UD tapes were produced using the same laboratory-scale processing route; consistent thermal, mechanical, and kinematic conditions were maintained during production. This approach allowed the observation of reinforcement–matrix interactions and production-induced defects without creating process-dependent bias between material systems.

### 2.2. UD Tape Production Process

UD thermoplastic composite tapes were produced using a continuous impregnation and consolidation line on a laboratory scale. The process consisted of fiber opening, fiber spreading, melt impregnation, consolidation with heated tools, and controlled cooling. During production, carbon fiber drawing tools were first stretched and spread open, thus ensuring uniform fiber distribution prior to resin impregnation. The molten PP matrix was then added to impregnate the fiber bundle under high temperature and pressure conditions. Key process parameters affecting belt quality included process temperature, line speed, and applied fiber tension. Changes in these parameters directly affected melt viscosity, impregnation efficiency, fiber alignment, and interfacial bonding. Insufficient temperature or excessive line speed led to resin non-impregnation, while incorrect tension control caused fiber waviness, fiber twisting, wrinkling, or local fiber breakage. After processing, the tape was consolidated and cooled to stabilize the microstructure, preserving the unidirectional architecture.

### 2.3. Defect Formation Due to Production

Production-related defects observed in the produced UD tapes have primarily been associated with local deviations in process stability and environmental interactions during processing. Interface separation and separation defects have been associated with insufficient impregnation pressure or temperature gradients across the tape width. Matrix-related defects, such as resin-rich or resin-poor areas, have resulted from irregular melt flow and variations in fiber packing density. Fiber-related defects, such as misalignment and wrinkling, have been caused by fluctuations in fiber tension and spreading efficiency. Additionally, air entrapment during cooling and contact with production guide elements, scratches, and environmental factors caused surface-level defects such as localized surface damage.

A representative defect environment was provided for the development and evaluation of the proposed visual defect detection framework with the defined production route and parameter sensitivity. Rather than eliminating these defects through comprehensive process optimization, this study deliberately focused on their systematic observation, classification, and detectability using vision-based methods under realistic laboratory production conditions.

### 2.4. Creation of Defect Taxonomy and Classification of Visual Characteristics

**Figure 1:**
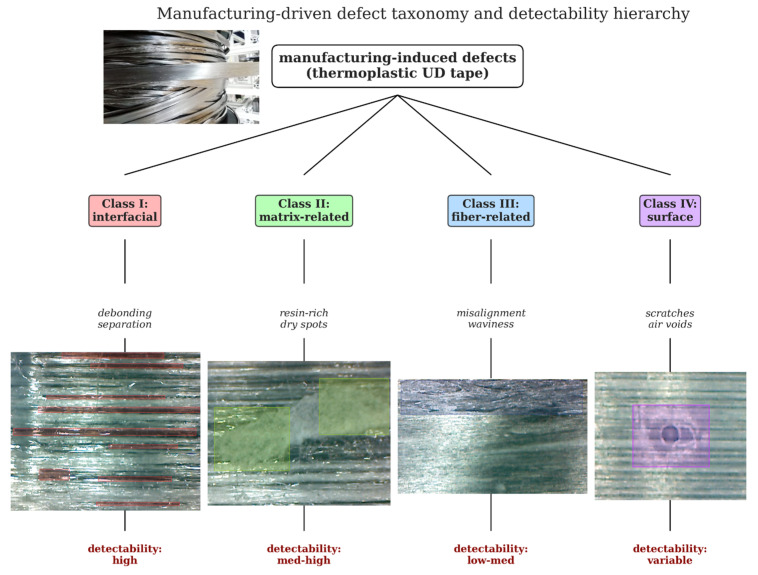
A production-oriented error taxonomy and relative visual perceptibility hierarchy.

Based on systematic observations made during the laboratory-scale production of PP–CF and PP–GF UD bands, defects arising from production have been divided into four main categories, as shown in Figure 1. The proposed taxonomy is driven by manufacturing physics and visual manifestation rather than post-consolidation damage mechanisms. This approach has established a direct link between process conditions, defect formation, and visual detectability, which is fundamental for practical in-line inspection systems.

The hierarchy shown in Figure 1 reflects the physical origin and visual manifestation of each defect class. Based on systematic observations during tape production, the four-class production-oriented defect taxonomy is detailed below, and the annotation label names used for YOLO training were structured consistently with these four defect categories to ensure coherent dataset preparation and model evaluation.

Class I: Interfacial (Interface bond failure and separation defects); surface defects are associated with insufficient bonding between the carbon fiber reinforcement and the polypropylene matrix. These defects are typically caused by insufficient impregnation temperature, insufficient pressure during consolidation, or local thermal gradients across the tape width.

Class II: Matrix (Matrix/resin-related defects); caused by irregular melt flow during impregnation and local variations in fiber packing density. Common symptoms include resin-rich areas, areas with resin deficiency, and areas with insufficient fiber wetting. These defects are strongly influenced by the process temperature and line speed, which determine melt viscosity and impregnation efficiency.

Class III: Fiber (Fiber-related defects); fluctuations in fiber tension, insufficient fiber spreading, or mechanical damage during processing are the causes. This category includes fiber misalignment, local waviness, wrinkling, and partial fiber breakage. Such defects alter the unidirectional architecture of the tape and can significantly reduce load transfer efficiency.

Class IV: Surface (Air entrapment and surface defects); surface-level defects caused by air bubbles and environmental interactions constitute the final category in the proposed taxonomy. These include void-related surface marks, scratches, abrasions, and dust and fibers originating from cooling and climate control, as well as local surface damage occurring during contact with guide components [1,35,39,40,45].

### 2.5. Relative Detectableness and Classification Rationale

In this context, the defect categories identified in the classification exhibit significant levels of visual detectability, directly influencing the choice of object detection over segmentation in this study. Based on experimental observations, the relative detection order was as follows: interface separation and detachment defects, matrix-related defects, fiber-related defects, and airborne or environmentally induced surface defects. This classification reflects the physical size and visual contrast of the defects, as well as their spatial continuity along the tape length.

By structuring defects according to their manufacturing origin and visual characteristics, the proposed taxonomy provides a practical framework for training vision-based inspection systems under realistic manufacturing constraints. Furthermore, it has established a common language for reporting and comparing manufacturing defects in continuous thermoplastic composite tapes.

### 2.6. Vision-Based Defect Detection Methodology and Experimental Setup

**Figure 2:**
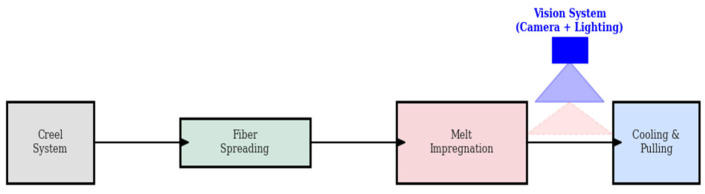
The laboratory-scale UD tape production line and a close-up view of the vision sensor positioned for inline monitoring.

To provide a physical context for the vision-based inspection framework, the laboratory-scale continuous impregnation and consolidation line used in this study, the camera and lighting positioning, and the region of interest used in image and video acquisition are shown schematically in Figure 2.

This schematic layout highlights key processing stages such as fiber dissolution and spreading, thermoplastic melt impregnation, consolidation, and cooling. The system is designed to reveal defects arising from production in a visually observable manner; image and video data were captured below the consolidation zone. Here, surface characteristics and defect indications have been stabilized for reliable visual inspection.

#### 2.6.1. Image and Video Data Acquisition

The visual data used in this study were obtained directly from real images captured during the production process, without the use of any synthetic data. High-resolution images were captured under consistent lighting conditions to determine surface appearance and defect morphology. Additionally, four short video sequences, each lasting approximately 30 min, were recorded. These sequences represent continuous production behavior and defect evolution along the belt length. The dataset was designed to intentionally reflect realistic laboratory conditions, including small variations in lighting, belt position, and surface texture, to enhance the robustness of the trained model.

The dataset used in the study consists of a total of 300 high-resolution macroscopic images obtained from the UD tape production line. The data set, which enables more stable tracking of the model’s validation metrics during the training process, has been randomly split in a 70:20:10 ratio, which is widely accepted in the literature to increase the model’s generalization ability and prevent overfitting. Rather than maximizing the dataset size, emphasis was placed on capturing different defect manifestations corresponding to the proposed defect taxonomy. This approach was consistent with practical production environments, where defect variety is often more critical than large sample volumes. The UD tape-specific augmentation strategy applied to these images is described separately in next section.

#### 2.6.2. Manufacturing-Aware Data Preparation and UD Tape-Specific Augmentation Strategy

The dataset was divided into training, validation, and test subsets using a stratified sampling strategy (stratified split dataset) to preserve the original class distribution across all subsets and avoid class imbalance bias in performance evaluation.

To enrich the training set, a combination of UD tape-specific albumentations and on-the-fly (online) augmentation was applied as a computationally efficient and physically consistent data expansion strategy. The UD tape-specific augmentation strategy developed in this study, together with the applied augmentation methods, is summarized in Table 1. All transformations were carefully constrained to preserve the original image aspect ratio and defect morphology, thereby avoiding any artificial geometric distortion. Rather than generating an excessively large synthetic dataset, this approach aimed to increase the effective diversity of a limited number of real production images, resulting in a compact yet physically representative augmented dataset suitable for reliable defect classification.

Manual defect annotation was carried out using the open-source web-based labeling platform makesense.ai [46]. Each defect example was labeled according to the four predefined classes mentioned above. Special attention was paid to maintaining consistency in annotation boundaries to reduce annotation noise and improve model generalization. The annotated dataset was then converted to a YOLO-compatible format for training and evaluation.

#### 2.6.3. Rationale for Object Detection Instead of Segmentation

The decision to use object detection instead of segmentation has led to a class-based performance analysis presented in the Results section, enabling both production realism and direct classification based on the visual characteristics of defects. Many defects arising from the production of continuous thermoplastic tapes exhibit slow transitions, partial overlaps, or elongated geometries that are difficult to distinguish without a lengthy labeling effort at the pixel level. Object detection has provided a pragmatic compromise by localizing defect regions while maintaining low annotation cost and computational load.

Bounding box annotations were chosen to support object detection rather than pixel-based segmentation. This choice was economically motivated by the visually scattered structure of several defect classes, overlapping defect boundaries, and the limited availability of high-resolution ground truth required for reliable segmentation.

Overall, the adopted vision-based methodology emphasizes practicality, scalability, and alignment with real production constraints over pursuing algorithmic complexity. This approach suggests the rapid implementation and adaptation of defect detection systems for continuous thermoplastic composite tape production environments.

#### 2.6.4. Object Detection Architecture and Training Procedure

The training and testing of the deep learning models were performed on an Apple Mac Mini M4 compact workstation. The system is powered by an Apple M4 chip featuring a 10-core CPU, a 10-core GPU, and a 16-core Neural Engine. Owing to the 16 GB Unified Memory architecture, the high memory requirements during model training (approximately 9 GB for YOLO11s) were satisfied without data transfer bottlenecks. All training processes were executed on the GPU using Python 3.10.19, the Ultralytics YOLO framework, PyTorch 2.9.1, and the Metal Performance Shaders (MPS) acceleration backend.

Lightweight YOLOv8 and YOLOv11 object detection architectures were employed due to their favorable trade-off between detection accuracy and computational efficiency. In addition, the most recent YOLO26s architecture, which eliminates the conventional Non-Maximum Suppression (NMS) step and enables end-to-end learning, was also evaluated [46]. YOLOv8 and YOLOv11 were utilized simultaneously as established benchmark models to provide a structured comparative baseline for evaluating YOLO26 within the same manufacturing context. This design enabled the assessment of YOLO26 against both a widely adopted mature reference model and a more recent optimized generation. The selection of compact models was motivated by the objective of achieving real-time or near real-time inspection capability in laboratory and industrial production line environments.

Model training was conducted through Python-based workflows executed via the command-line interface. Transfer learning was applied by initializing the networks with pre-trained weights and subsequently fine-tuning them on the custom composite tape defect dataset. To ensure stable convergence under limited data conditions, training hyperparameters were carefully selected, and UD tape-specific augmentation strategy techniques were employed to enhance robustness against minor visual variations. Table 2 summarizes the main training hyperparameters used during model fine-tuning, including input resolution, batch size, number of epochs, optimization strategy, and confidence thresholds.

### 2.7. Evaluation Metrics and Confidence Threshold Determination

Model performance was evaluated both qualitatively and quantitatively based on the detection confidence scores and the visual inspection of the predicted bounding boxes. It is expected that the obtained confidence levels will be consistent with the dominant defect classes. For instance, lower confidence scores are anticipated for fiber-related defects and surface-level environmental defects, which inherently reflect their subtle visual characteristics.

For the quantitative assessment of model performance, the confusion matrix and its associated metrics, which are widely used in object detection problems, were adopted. The confusion matrix enables a systematic analysis of the model’s correct and incorrect classification behavior. In this context, the following definitions are used [29,47,48]:

- TP (True Positive): A region that truly contains a defect and is correctly detected as defective by the model.
- TN (True Negative): A region that does not contain a defect and is correctly classified as defect-free by the model.
- FP (False Positive): A region that does not contain a defect but is incorrectly detected as defective by the model.
- FN (False Negative): A region that truly contains a defect but is missed by the model and incorrectly classified as defect-free.

Based on these fundamental definitions, the model performance is evaluated using the following standard metrics.

Precision, which represents the proportion of regions detected as defective by the model that are actually defective, is defined as:

(1) $$Precision=\frac{TP}{TP+FP}$$

This metric directly reflects the impact of false positives (FP) on the model outputs [49]. Recall, which indicates how many of the actual defects that should be detected are successfully identified by the model, is defined as:

(2) $$Recall=\frac{TP}{TP+FN}$$

Recall is of critical importance in manufacturing environments, since a high FN rate directly corresponds to missed defects and therefore compromises quality assurance and product reliability [49,50]. Because Precision and Recall often exhibit a trade-off relationship, the F1-score metric, which provides a balanced evaluation of both, is also employed. The F1-score is defined as the harmonic mean of Precision and Recall:

(3) $$F1=2\cdot \frac{Precision\cdot Recall}{Precision+Recall}$$

The F1-score is particularly important in defect detection problems, as it simultaneously accounts for both false positives and false negatives. In this study, the F1-score is employed to evaluate whether the model exhibits balanced and reliable decision behavior under manufacturing conditions. In human-in-the-loop inspection scenarios, a high F1-score indicates that the model can be effectively used as a pre-screening tool. Average Precision (AP) represents the area under the Precision–Recall (PR) curve (Area Under Curve, AUC) and provides a comprehensive measure of the model’s overall detection performance across different confidence thresholds. The mean Average Precision (mAP) is defined as the average of the AP values computed over all defect classes and serves as a principal metric for summarizing the overall performance of multi-class object detection systems. In this study, the mAP@0.5 metric is adopted, where “0.5” denotes the Intersection over Union (IoU) threshold required for a detection to be considered correct. The IoU is defined as the ratio of the intersection area between the predicted bounding box and the ground truth bounding box to the area of their union [51,52]:

(4) $$IoU=\frac{Area(Intersection)}{Area(Union)}$$

(5) $$\mathrm{mAP}=\frac{1}{N}\sum_{i=1}^{N}AP_{i}$$

When the IoU value exceeds a predefined threshold, the detection is considered correct. In order to evaluate the suitability of the model for manufacturing environments, inference time and Frames Per Second (FPS) are also taken into account in addition to accuracy-based metrics. Inference time refers to the duration between providing a single image to the model and obtaining the corresponding prediction results, whereas FPS represents the number of images that the model can process per second [20,29,53]. These metrics are of critical importance for the integration of the system into real-time or near-real-time in-line inspection applications. Moreover, they enable a consistent comparison with the performance criteria reported in the literature for machine-learning-based and non-destructive testing approaches applied to damage detection and quality control in composite materials.

The loss functions used during model training consist of three main components:

- Objectness loss: Determines whether the predicted bounding box actually contains an object (defect) and enables the model to learn the distinction between foreground and background.
- Localization loss (box regression loss): Measures the geometric error between the predicted bounding box and the ground truth, and is applied only when an object is detected.
- Classification loss: Ensures the correct assignment of the predicted defect to its corresponding category [53,54,55].

In addition, to ensure lower model complexity, reduced hardware requirements, and easier integration into production lines, the s and n variants of the YOLO architectures were preferred. Model performance was quantitatively evaluated using the mAP, Precision, Recall, and F1-score metrics calculated based on the TP, FP, TN, and FN parameters described above [20,29].

In this study, compact architectures capable of providing reasonable accuracy levels at low computational cost were deliberately selected. In this way, the models offer an economically efficient and practically acceptable detection performance while maintaining strong industrial applicability. Finally, model performance was assessed not only through numerical metrics but also by visual analysis of TP, FP, and FN samples. This qualitative evaluation makes it possible to understand the model’s decision behavior under manufacturing conditions, to identify the defect classes in which it performs strongly or weakly, and to interpret the physical origins of incorrect predictions. By complementing metric-based evaluation, this approach demonstrates the practical adequacy and production relevance of the proposed visual inspection framework.

## 3. Results and Discussion

The physical defects illustrated in Figure 1 were evaluated using a process-driven defect taxonomy and a visual classification approach based on production-oriented observations conducted during UD tape manufacturing, as summarized in Table 3. This taxonomy jointly considers the origin of defects, their dominant manufacturing mechanisms, visual characteristics, and relative detectability levels, thereby providing an integrated interpretation of defect formation from both physical and perceptual perspectives.

The results indicate that interfacial debonding and matrix-related defects are visually more prominent, whereas fiber alignment deviations and surface-level environment-induced defects are inherently more difficult to perceive due to their low visual contrast. In this respect, the proposed classification framework not only describes defect types but also provides a physically meaningful and interpretable basis that supports production-oriented quality assessment and automated visual inspection strategies for UD tape manufacturing.

In this context, Table 3 summarizes the proposed manufacturing-driven defect taxonomy for continuous UD thermoplastic composite tapes, including defect origin, visual characteristics, dominant process drivers, and relative detectability. This table forms the basis of a unified reference framework employed in the subsequent vision-based inspection analysis.

### 3.1. Defect Detection Performance Across Defect Classes

**Figure 3:**
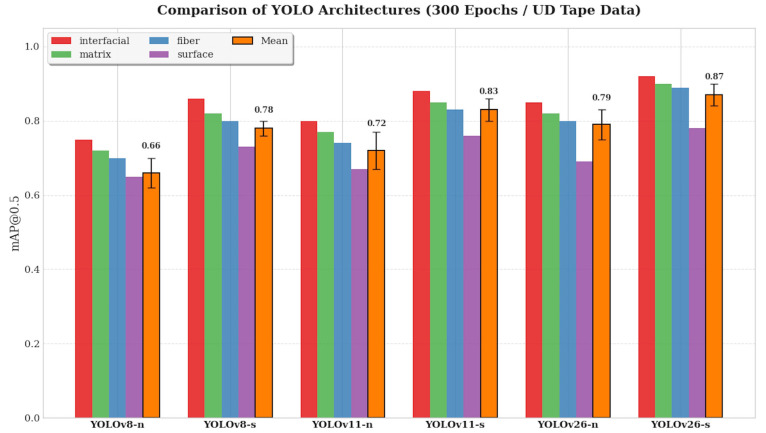
Mean mAP@0.5 comparison of YOLOv8, YOLOv11 and new YOLO26 (with n and s variants, 300 epochs by UD Tape-specific augmentation strategy).

The average mAP@0.5 values obtained for different model scales of the YOLOv8, YOLOv11, and the newly introduced YOLO26 families are compared in Figure 3. These results present the class-wise mAP@0.5 performance of different YOLO model families and capacity levels (n, s), all trained for 300 epochs using and Developed in this study UD Tape-specific augmentation strategy.

In this context, it has been observed that even lightweight YOLO models at nano (n) and small (s) scales can achieve high success in detecting manufacturing defects. The consistent performance increase observed from YOLOv8 to YOLOv11 and then to YOLO26 reveals that architectural advancements have significantly improved detection accuracy without the need for larger and more complex models.

This observation supports the suitability of compact network architectures for real-time inspection in continuous thermoplastic tape production lines. Model performance was evaluated using Precision, Recall, and mAP@0.5 metrics, while inference speeds were recorded in milliseconds (ms) on the Apple M4 processor using the Metal Performance Shaders (MPS) acceleration framework. The comparative results are presented in Table 4.

In addition to detection accuracy, computational efficiency was evaluated using approximate FLOPs, parameter count, inference time, and FPS. The YOLO26 models benefit from an end-to-end architecture that embeds the NMS post-processing step into the network itself, thereby reducing latency and improving real-time performance. Moreover, ongoing optimization efforts aim to further enhance compatibility and efficiency on Apple Silicon-based Mac processors. making it a promising candidate for compact and energy-efficient industrial deployment.

The newly trained YOLO26-s model, which exhibits high computational efficiency, demonstrated differentiated detection behavior across the four defined defect categories, reflecting their distinct visual and physical characteristics.

Trained for 300 epochs using UD tape-specific on-the-fly data augmentation together with a physically constrained albumentations pipeline and exclusively real production images, the model demonstrated stable, physically meaningful, and class-consistent performance in detecting manufacturing-induced defects.

**Figure 4:**
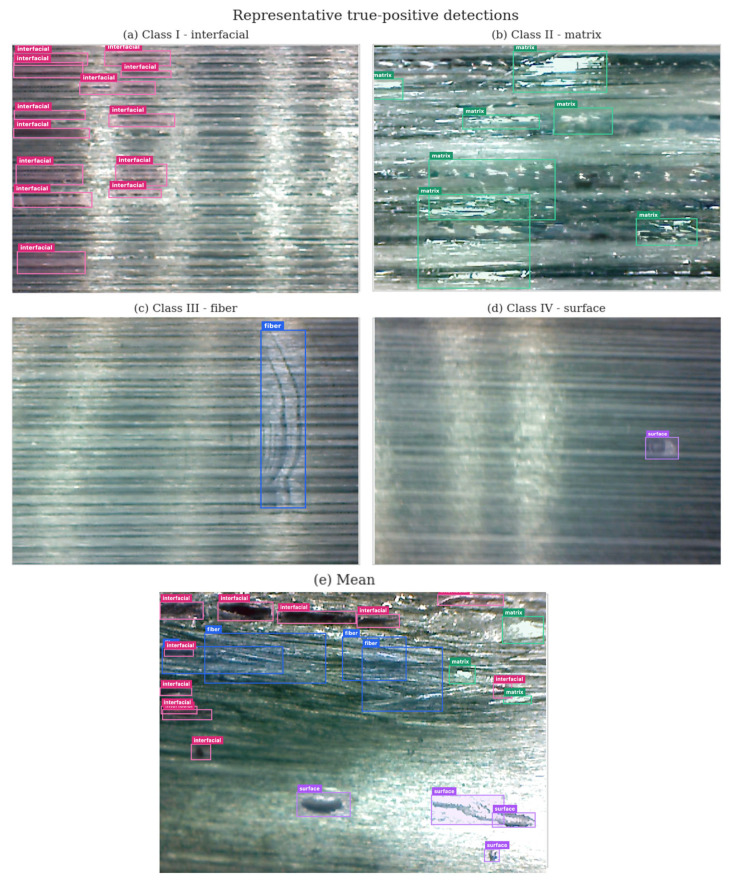
Representative true-positive detection examples for each defect category.

Without relying on synthetic data, the quantitative detection performance is summarized in Table 5 through class-wise performance metrics corresponding to the defined YOLO labels across the validation folds. Furthermore, Figure 4 presents representative true-positive detection react examples for each defect category, illustrating the diversity of defect morphologies encountered during tape manufacturing.

Rather than applying aggressive confidence thresholds, the detection outputs were analyzed in accordance with practical inspection requirements. In this context, early identification of potential defect regions was prioritized over strict minimization of false positives. This strategy supports the integration of the proposed framework into human-in-the-loop or hybrid inspection systems, where preliminary screening and expert validation can be effectively combined. The class-wise evaluation is discussed below.

Class I—Interfacial separation and debonding defects exhibited the highest detection confidence among all categories. These defects typically present long and continuous geometries aligned with the fiber direction, forming clear and high-contrast boundaries that are well captured by bounding-box-based object detection. Visually, interfacial defects appear as discontinuous regions, longitudinal separations, or localized voids oriented along the fiber direction. Owing to their relatively high contrast and geometric continuity, these defects exhibit sharp visual boundaries, making them the most readily detectable defect class using object detection approaches. Consequently, interfacial debonding defects achieved the highest confidence levels across all categories.

Class II—Matrix-related defects exhibited moderate to high detection confidence in clustered regions. Variations in surface texture and gloss associated with resin-rich and resin-starved areas enabled visual discrimination. However, specular reflections and diffuse boundaries occasionally led to partial or fragmented detections. Despite these challenges, such defects were reliably identified as anomalous regions, supporting their suitability for early-stage quality monitoring.

Visually, matrix-related defects manifest as localized changes in surface texture and brightness on the tape surface and are typically characterized by diffuse boundaries. Although they are more challenging to detect than interfacial defects, resin-related anomalies achieved moderate to high detectability due to their distinctive surface appearance under controlled illumination conditions.

In contrast, concerning Class III, fiber-related defects exhibited lower and more variable detection confidence. Subtle deviations in fiber alignment, local waviness, dispersed wrinkles, and fuzz balls affect the tape architecture but do not generate strong contrast variations. Visually, fiber-related defects manifest as fine geometric distortions rather than sharp intensity changes. It was observed that interfacial and matrix-related defects are generally more detectable than fiber-related and surface-level environmental defects. Consequently, identifying such subtle variations is inherently challenging for vision-based systems, particularly when defect severity is low or when defects are distributed over large areas. This category therefore represents one of the most critical limitations of automated inspection based solely on surface imaging. In particular, when defects spread gradually over wide regions, the model occasionally failed to sufficiently detect low-severity fiber misalignment. This behavior highlights an intrinsic limitation of surface-based visual inspection for capturing fine-scale architectural deviations in unidirectional composites.

Finally, concerning Class IV, air entrapment, dust contamination, foreign objects, and environmentally induced surface defects represent a multi-factor category for automated detection. Such defects are typically small-scale, randomly distributed, and often visually similar to benign surface features. Their stochastic nature and low contrast, together with visual artifacts caused by surface texture or illumination effects, make consistent detection using object detection models challenging. As a result, this class exhibited relatively lower detection reliability and higher sensitivity to imaging conditions. Nevertheless, identifying these factors remains important, particularly in automated production environments, as they may influence material flow behavior and consolidation quality. These results quantitatively confirm the hierarchy of visual detectability derived from the production-oriented observations. Despite these challenges, the model successfully localized prominent surface anomalies, demonstrating its potential as a screening tool rather than as a definitive classifier for this defect category.

**Figure 5:**
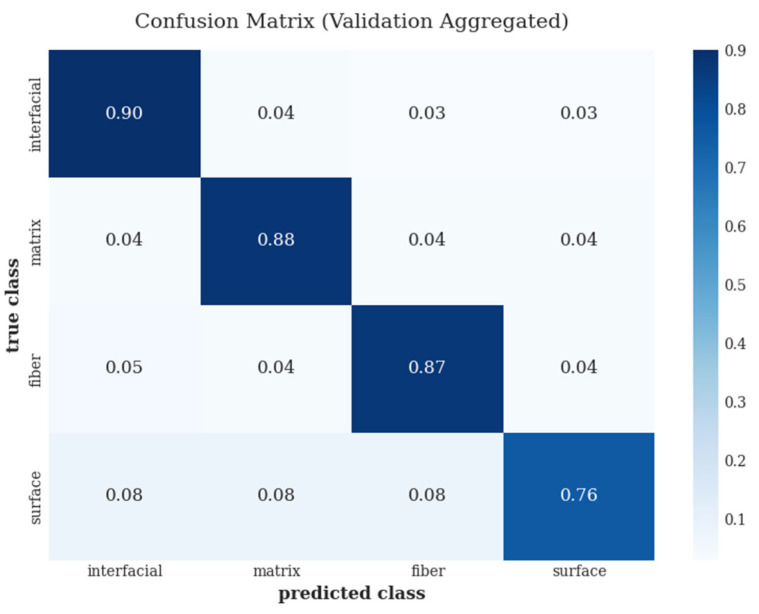
Confusion matrix summarizing the class-wise prediction performance for manufacturing-induced defect detection in continuous UD thermoplastic composite tapes.

Furthermore, beyond aggregate performance metrics, the confusion matrix shown in Figure 5 was analyzed to further investigate class-wise misclassification behavior. In the matrix, rows correspond to the ground-truth defect classes, while columns represent the predicted classes. The values denote validation results aggregated across folds, highlighting the dominant misclassification patterns between visually similar defect categories. This analysis provides additional insight into the strengths and limitations of the proposed visual inspection framework at the class level.

As shown in Figure 5, interfacial defects were predominantly classified correctly and exhibited the lowest level of confusion with other defect types. Misclassifications were more pronounced between matrix-related and fiber-related defects, reflecting the overlap in their visual features and their diffuse surface manifestations. Surface-level air- and environment-induced defects exhibited the highest confusion rates and were occasionally misidentified as matrix-related anomalies, indicating the visual similarity between these categories. These trends are consistent with the relative detectability hierarchy derived from the production-oriented observations and the class-wise performance metrics.

The experimental results further revealed a significant difference in defect detectability between the two material systems, governed by their optical properties. As reported in optical characterization studies of composite materials, carbon fibers exhibit high optical absorption and form sharp contrast boundaries against the semi-transparent polypropylene (PP) matrix. This increased contrast facilitates feature extraction for convolution-based detection networks such as YOLO. In contrast, glass fibers and the PP matrix possess similar refractive indices and optical transparency, leading to a “transparency masking” effect. This phenomenon, discussed in the context of transparent composite manufacturing and optical homogenization, reduces the pixel intensity gradients required for reliable edge detection [22,34,39,56].

**Figure 6:**
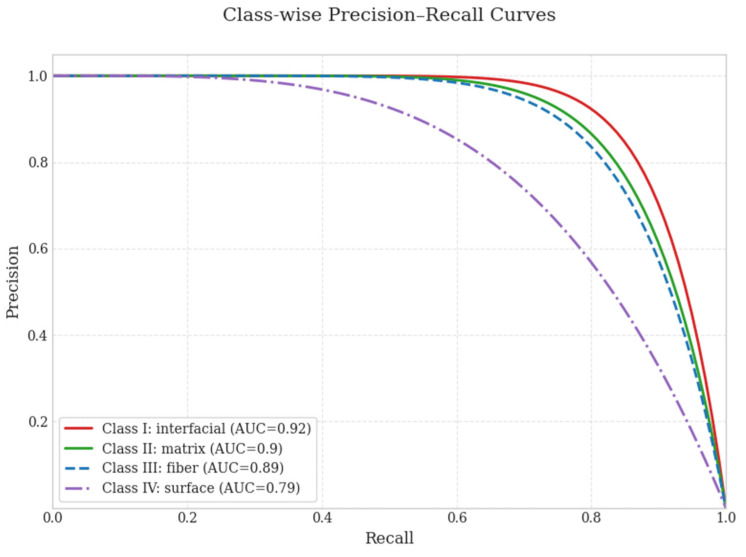
Precision–Recall trade-off for each defect class.

Consequently, fiber-related defects (Class III) in PP–GF tapes tend to blend into the background texture, providing a physical explanation for the lower mAP values observed for this defect class compared to PP–CF tapes. Figure 6 presents the class-wise Precision–Recall curves, highlighting differences in detection behavior and the trade-offs in confidence levels among the defect categories.

The hierarchy observed in the detection performance indicates that interfacial debonding defects, matrix-related defects, fiber-related defects, and surface-level environmental defects directly correspond to the fundamental physical manifestations of each defect type. Defects that produce macroscopic discontinuities or localized material separation are naturally easier to detect using object detection frameworks, whereas defects characterized by gradual geometric distortions or low visual contrast introduce intrinsic challenges for reliable detection.

### 3.2. Practical Implications for Manufacturing Inspection

**Figure 7:**
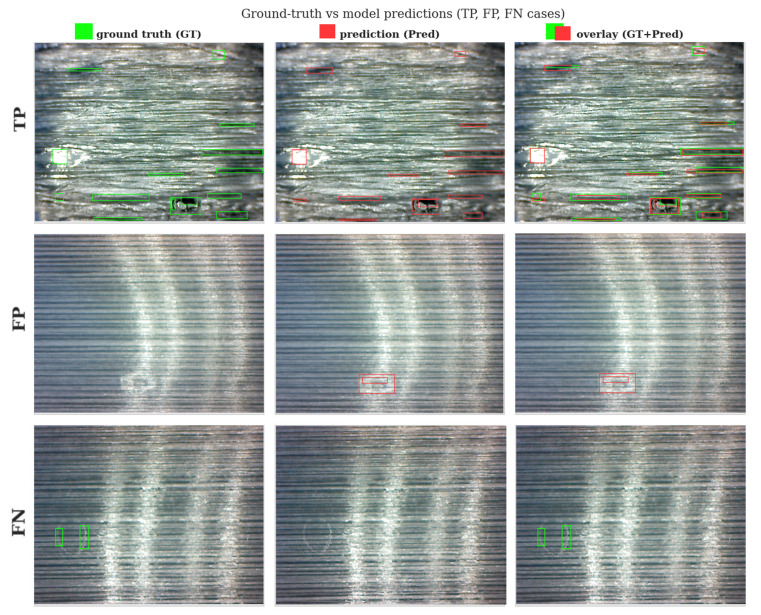
Region-based representations of TP, FP, and FN cases for object detection on UD tapes.

To support the qualitative interpretation of model behavior under realistic production conditions, representative examples of correct and incorrect detections are presented in Figure 7, while the corresponding quantitative results are listed in Table 6. These include true positives (TP), false positives (FP), and false negatives (FN) for the different defect categories, highlighting the typical success and failure modes of the proposed detection framework. This combined qualitative and quantitative analysis provides practical insight into how the model operates in real manufacturing environments and clarifies its strengths and limitations across different defect classes.

Representative detections are shown as follows: the left column displays the ground-truth bounding boxes, the middle column presents the model predictions with confidence scores, and the right column compares the predictions overlaid on the original images. The first row corresponds to interfacial debonding defects, the second row to matrix-related defects, and the third row to fiber-related and surface-level defects.

The counts of TP, FP, and FN presented in Table 6 reflect the relationship between the model’s prediction success and the manufacturing-induced physical and optical properties of the UD tapes. The FP cases in matrix and surface categories are primarily associated with specular reflections and diffuse boundaries on the tape surface. Following an ‘early-warning’ philosophy, the confidence thresholds were optimized to prioritize identifying potential defect regions over strictly minimizing false positives, as the industrial cost of a missed defect (FN) far outweighs that of checking a false alarm. Regarding false negatives, the higher FN rates in fiber and surface classes are attributed to ‘transparency masking’ effects, particularly in PP-GF systems where similar refractive indices between the fiber and matrix limit the pixel intensity gradients required for reliable detection. This analysis confirms that the detection performance is fundamentally governed by the fiber–matrix optical contrast established during tape manufacturing.

**Figure 8:**
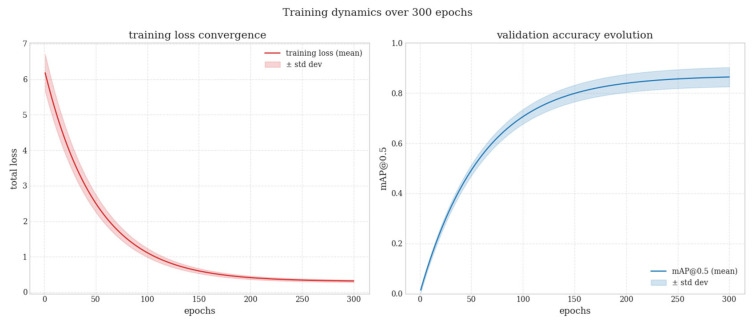
Training/Validation performance over 300 epochs.

As illustrated in the figure, interfacial debonding defects are generally detected with high confidence and accurate localization, whereas matrix-related defects occasionally exhibit partial or fragmented detections due to their diffuse boundaries. Fiber-related and surface-level defects show a higher frequency of false negatives and false positives, reflecting their low visual contrast and stochastic surface appearance. These qualitative observations are consistent with the previously reported class-wise performance trends. Finally, Figure 8 presents the convergence behavior of the validation curves through the evolution of the loss functions and the mAP values over the training process.

Training and validation performance trajectories over 300 epochs are summarized as mean ± standard deviation across three independent training repetitions conducted under identical hyperparameter settings but different random seed initializations. The rapid and stable decrease in training loss indicates that the model achieves reliable convergence during the learning process. The steady increase in the validation mAP@0.5 with increasing epochs, followed by a plateau after approximately the 200th epoch, demonstrates that the generalization capability of the model stabilizes and that no evident overfitting behavior is observed. The narrowing of the standard deviation bands further indicates consistent and repeatable performance across different folds, confirming that the data augmentation strategy effectively enhances training stability and generalization despite the limited dataset size.

When examining the effect of reinforcement type on defect visibility (PP–CF vs. PP–GF), a critical observation emerges from the experimental analysis: a pronounced difference in visual detectability arises in computer vision tasks, governed by the optical properties of the reinforcing fibers.

For carbon fiber-reinforced tapes (PP–CF), the high optical contrast between the opaque black carbon fibers and the semi-transparent polypropylene matrix facilitates feature extraction for the learning architecture. Defects such as voids, interfacial separations, and fuzz balls generate sharp intensity gradients, resulting in higher detection confidence and more precise bounding box localization.

In contrast, for glass fiber-reinforced tapes (PP–GF), the glass fibers exhibit refractive indices and color characteristics similar to those of the semi-crystalline PP matrix. This lack of optical contrast leads to a “masking effect”, particularly for Class III (fiber-related) defects. Subtle waviness or misalignment in PP–GF tapes tends to blend into the background texture, making localization difficult without specialized illumination schemes such as polarized or backlighting configurations.

This material-dependent behavior indicates that future industrial inspection systems may require adaptive illumination strategies tailored to the specific optical characteristics of the fiber–matrix system. Such adaptation will be essential to ensure robust and reliable defect detection across different reinforcement types in continuous thermoplastic composite tape manufacturing.

Importantly, the reduced detectability of fiber-related and surface-level defects should not be interpreted solely as a limitation of the employed deep learning architecture. Rather, it reflects the intrinsic difficulty of capturing three-dimensional fiber architecture and microscale surface damage using two-dimensional surface images. Addressing these limitations may require complementary sensing modalities, such as directed or structured illumination, multi-view imaging, or the integration of process signals, which lie beyond the scope of the present study.

While previous AFP and ATL oriented studies have predominantly focused on geometrical defects induced during surface placement on consolidated composite parts, the present results demonstrate that defect detectability at the tape production stage is strongly governed by the optical interaction between the reinforcement and the thermoplastic matrix [20,31,32,33,34,42]. This distinction becomes particularly evident when comparing PP–CF and PP–GF systems. Whereas AFP-focused inspection systems emphasize defect localization and post-placement visualization, our findings indicate that defect detectability is fundamentally constrained by the fiber–matrix optical contrast established during tape manufacturing. This explains why certain defect classes remain challenging to detect even with advanced detection frameworks.

From a manufacturing perspective, the results show that a compact object detection model can provide meaningful and practically applicable insight into tape quality under realistic laboratory-scale production conditions. The rapid decrease in the training loss and its convergence to a stable plateau at later epochs indicate that the model effectively learned the dataset without exhibiting signs of overfitting. Moreover, the low standard deviation values observed across the 5-fold cross-validation confirm that the learning process is robust against different data partitions.

Together with the convergence behavior observed in both training and validation losses, the achieved accuracy and confidence levels in the range of 0.84–0.94 suggest that the proposed approach provides sufficient reliability for manufacturing-oriented inspection scenarios. In particular, when integrated into human-in-the-loop inspection strategies, the model can effectively support the identification of suspicious regions, guide further detailed examination, and enable timely adjustment of process parameters when necessary.

Recent vision-based composite inspection studies have predominantly focused on Automated Fiber Placement (AFP), Automated Tape Laying (ATL), and fully consolidated composite components, and are therefore typically conducted under controlled imaging conditions using large, well-curated datasets [12,13,28,29,31,43]. YOLO-based object detection architectures have demonstrated high accuracy and real-time capability in detecting surface defects such as voids, overlaps, wrinkles, and foreign inclusions in AFP and ATL processes [12,21,29,30,31,34,41]. These studies mainly target geometrical defects characterized by sharp visual contrast and well-defined boundaries, thereby achieving high detection confidence and emphasizing algorithm-oriented performance optimization [11,28,45].

In contrast, the present study addresses continuous thermoplastic UD tape manufacturing prior to downstream placement or consolidation. In this stage, defects are typically associated with diffuse boundaries, gradual transitions, and a strong dependence on process-induced material heterogeneity [2,16,37,38,39,40]. Unlike inspections performed on fabrics or fully consolidated laminates, tape-level defects such as incomplete impregnation, subtle fiber misalignment, and interfacial separation are visually less prominent and are significantly more sensitive to illumination conditions and surface texture [10,35,39].

Similar challenges have also been highlighted in broader reviews of machine-learning-based composite inspection, which emphasize the limited transferability of models trained under idealized conditions to realistic manufacturing environments [5,6,18,28].

Moreover, while many studies aim to maximize detection accuracy through deeper architectures, attention mechanisms, or large-scale datasets [5,6,17,19,21,29,34,41], the present work adopts a different design philosophy. By combining a manufacturing-driven defect taxonomy with a lightweight detection model, this study prioritizes interpretability, scalability, and practical applicability under data-limited laboratory conditions. This perspective is well aligned with recent calls for physically informed defect definitions and representative defect datasets to support digital manufacturing and closed-loop quality control in composite production [11,23,28,32,37,38,39,41,42,43].

The comparative analysis demonstrates that the main contribution of this study lies not in competing on absolute detection accuracy, but in showing that production-induced defects in continuous thermoplastic UD tapes can be reliably, economically, and compactly detected using object detection models. In this sense, the proposed approach is positioned as a complementary and practice-oriented contribution to the vision-based composite inspection literature.

### 3.3. Limitations and Future Research Directions

While the proposed approach demonstrates clear applicability, several limitations must be acknowledged. The dataset size has been deliberately limited to reflect laboratory-scale production, potentially restricting model generalization to different material systems or industrial-scale processes. Furthermore, relying on surface images limits the detectability of subsurface defects and fine-scale fiber architecture variations.

Future work will focus on expanding the dataset under different process conditions, incorporating temporal information from continuous video streams, and exploring multimodal inspection strategies. In particular, the integration of thermal cameras and multi-view imaging systems capable of monitoring the tape from both the upper and lower surfaces may improve the detection of low-contrast, surface-sensitive, and process-related defect signatures. Another key objective will be to develop low-cost, high-efficiency inspection configurations that improve robustness while preserving industrial feasibility. The combined use of process parameters, RGB imaging, thermal data, dual-surface camera monitoring, and real-time feedback mechanisms represents a promising pathway toward robust and economically scalable closed-loop quality control systems in thermoplastic composite tape production.

## 4. Conclusions

This study presented a manufacturing-oriented framework for the characterization and visual detection of defects in continuous unidirectional polypropylene–carbon fiber and polypropylene–glass fiber thermoplastic composite tapes. Based on direct production observations, a practical four-class defect taxonomy was established, comprising interfacial, matrix-related, fiber-related, and surface/environment-induced defects. In this respect, the study provides a process-relevant classification structure that links defect formation mechanisms to their visually observable manifestations in UD tape manufacturing.

To evaluate the feasibility of automated inspection, lightweight YOLO-based object detection models were comparatively assessed under limited-data conditions. A key strength of the proposed framework is that augmentation was not applied indiscriminately; instead, it was deliberately constrained by the directional and morphological realities of continuous UD tape production in order to preserve manufacturing realism and avoid physically implausible transformations. This point is particularly important for UD systems, in which defect appearance is strongly coupled to process direction, local material architecture, and production-induced visual patterns. Despite the limited number of original production images, this physically informed and manufacturing-aware augmentation strategy enabled robust model training and effective defect discrimination, with confidence values reaching up to 0.94 and YOLO26-s providing the strongest overall performance among the evaluated lightweight models.

The results further showed that defect detectability was strongly class-dependent. Interfacial and matrix-related defects were identified more successfully because of their clearer geometric discontinuities and stronger contrast, whereas fiber-related and low-contrast surface anomalies remained more challenging due to their weaker optical salience and more subtle visual signatures. These findings indicate that visual inspection at the UD tape stage can support early defect screening before downstream consolidation or conversion into higher-value components, thereby offering practical benefits in terms of waste reduction, process stability, and manufacturing repeatability.

At the same time, the present results should be interpreted within the limits of the adopted dataset and imaging conditions. The relatively small dataset size, laboratory-scale validation, and reliance on surface-visible defect manifestations constrain broader industrial generalization. Future work should therefore focus on larger and more heterogeneous datasets, external validation under different production conditions, and multimodal inspection strategies such as thermal imaging and top–bottom camera configurations. The integration of imaging outputs with process parameters and real-time feedback mechanisms may further support the development of low-cost, scalable, and closed-loop quality control systems for thermoplastic composite tape manufacturing.

## Figures and Tables

**Table 1:** UD Tape-Specific Augmentation and Albumentation parameters.

Augmentation Parameters	Value	Description
Mosaic	0.0	Off. (It creates spurious defect neighborhoods that do not exist in actual production. It disrupts tape continuity and is physically meaningless for UD tape.)
Scale	0.1	Limited scaling to simulate minor camera distance variations.
Flip Left-Right	0.15	Rotates the image (Off in Albumentation). Simulates lateral orientation variation across tape width.
Translate	0.02	Shifts the image horizontally/vertically by ±2%.
HSV-Hue	0.0	Off. (It artificially distorts PP-CF/PP-GF contrast.)
HSV-Saturation	0.1	Mild lighting-induced saturation variation.
HSV-Value	0.15	Controlled brightness variation to emulate illumination changes.
Mixup	0.0	Off. (It artificially alters the real tape texture.)
Flip Up-Down	0.0	Off. (In UD conveyor belts, the production direction is unidirectional. This distorts the actual production conditions.)
Perspective	0.0	Off. Affine distortions.

**Table 2:** Main training hyperparameters used for fine-tuning the object detection models employed in this study.

Hyperparameter	Value (This Study)	Practical Interpretation in Manufacturing
Model architecture	Ultralytics YOLOv8, v11 and new YOLO26 (variants: s, n)	Lightweight structure suitable for real-time or near real-time inspection
Input image size	640p	Preserves surface texture while maintaining computational efficiency
Batch size	16 (depending on GPU/CPU memory)	Balance between convergence stability and hardware limits
Epochs	300	Ensures sufficient convergence for limited experimental datasets.
Learning rate	0.01/0.001 (default scheduler) & (with warm-up + cosine scheduler strategy)	Stable optimization without overfitting risk. This provides stable convergence, especially for limited datasets.
Optimizer	AdamW/SGD (default YOLO) (Momentum: 0.9, Weight Decay: 0.0005)	SGD enables stable and reliable learning of rare defects without being affected by noise, by reducing overfitting in small datasets.
IoU threshold	0.5	Standard value for bounding box correctness in object detection
Confidence threshold (lab)	0.5–0.6	Filters weak detections; suitable for controlled experimental evaluation
Confidence threshold (production)	0.20–0.30 (with operator supervision)	Lower threshold to minimize false negatives; early-warning philosophy
Non-maximum suppression (NMS)	Enabled (default YOLO)	Prevents multiple overlapping detections for the same defect
Transfer learning	Pretrained COCO weights	Strongly recommended and practically mandatory for small experimental datasets; ensures faster convergence and more robust training behavior.
Data augmentation	Scale, Flip, Brightness, Contrast, Noise	Applied to increase dataset variability and prevent overfitting. Improves robustness to lighting and surface texture variations
Training data type	Real experimental images only	No synthetic or simulated data used; full manufacturing realism
Inference mode	Object detection	(not segmentation) Faster annotation, robust for diffuse defect boundaries

**Table 3:** Manufacturing-driven defect taxonomy for UD tapes, including defect origin, visual characteristics, dominant process drivers, and relative visual detectability.

Defect Class	Subtypes	Physical Formation Mechanism	Production Process	Visual Characteristics	Relative Detectability
Class I: interfacial	Debonding, Longitudinal splitting, Gap	Poor wetting, thermal mismatch, insufficient consolidation pressure, impregnation defects, and localized cooling.	High temperature (T) ↑, low impregnation pressure, increased processing speed. ↑	High contrast, sharp edges, and continuous void lines along the fiber direction.	High
Class II: matrix	Resin-rich areas, Dry spots	Irregular melt flow, permeability variations, viscosity fluctuations, and fiber opening.	T incorrect, speed ↑	Variations in surface gloss (specular reflection), texture changes, diffuse boundaries, surface texture differences, and blurred edges.	Medium–High
Class III: fiber	Misalignment, Waviness, Blurring	Tension fluctuations, mechanical jamming, and fiber spreading defects.	Tension variation, spreading error.	Low contrast, subtle geometric deviations, fine texture variations without sharp intensity transitions, waviness along the fiber direction, and line distortion.	Medium
Class IV: surface	Scratches, Air pockets, Surface marks, Foreign objects	Handling, cooling, contact with rollers, entrapped air, and surface contamination.	Guiding and contact conditions, process control, working environmental conditions, ventilation.	Stochastic, geometric irregularities that are generally small-scale and distinct from the fiber texture; point-like spots, fine scratches, and random distribution.	Variable

**Table 4:** Performance list of YOLOv8-s, YOLOv11-s, YOLO26-s model architectures.

Metric	YOLOv8-s	YOLO11-s	YOLO26-s (New)	Notes
Architecture	CNN + NMS	Improved CNN	NMS-Free End-to-End	YOLO26 eliminated the post-processing computational overhead.
Precision(P)	0.82 ± 0.04	0.87 ± 0.03	0.90 ± 0.02	YOLO26 minimizes the “ghost box” (false alarm) problem.
Recall(R)	0.75 ± 0.04	0.81 ± 0.04	0.85 ± 0.03	Its end-to-end architecture enables better detection of overlapping and intertwined defects.
F1-score	0.78	0.78	0.87	More stable detection performance.

**Table 5:** Class-wise YOLO26s model performance metrics.

Class ID	“YOLO Label”	Defect Class Description	mAP@0.5 (Mean ± Standard Deviation)	Precision (P)	Recall (R)	F1-Score	Notes
Class I	Inter- facial	Interfacial separation and debonding defects	0.92 ± 0.02	0.92 ± 0.02	0.90 ± 0.03	0.91	High contrast
Class II	matrix	Matrix-related defects	0.90 ± 0.03	0.91 ± 0.03	0.88 ± 0.04	0.89	Diffuse boundaries
Class III	fiber	Fiber-related defects	0.89 ± 0.03	0.89 ± 0.03	0.87 ± 0.04	0.88	Fine scale, requires multi-modal analysis
Class IV	surface	Air and environment induced surface defects	0.79 ± 0.04	0.86 ± 0.04	0.76 ± 0.05	0.81	Small-scale
Mean	-	Overall	0.87 ± 0.03	0.90 ± 0.02	0.85 ± 0.03	0.87	-

**Table 6:** Counts of TP, FP, and FN for each defect class evaluated on the validation dataset.

Defect Class	YOLO Label	TP	FP	FN
Interfacial debonding	interfacial	99	9	11
Matrix-related defects	matrix	88	9	12
Fiber-related defects	fiber	79	9	11
Surface-level defects	surface	38	7	13

## Data Availability

The data presented in this study are available on request from the corresponding author due to the fact that the data supporting the findings of this study were generated under TÜBİTAK-funded research projects and are therefore subject to institutional data protection policies.
